# Dual-Mode Teaching in the Language Classroom: Reconciling the Pandemic, Equity, and the Future of Quality Language Teaching Pedagogy

**DOI:** 10.1007/s40841-022-00258-z

**Published:** 2022-08-03

**Authors:** Vincent Ieni Olsen-Reeder

**Affiliations:** Tauranga Moana Te Arawa, Te Herenga Waka – Victoria University of Wellington, Wellington, Aotearoa New Zealand

**Keywords:** Hybridisation, Language teaching, Language pedagogy, Online pedagogy

## Abstract

The COVID-19 pandemic sent New Zealand universities into crisis, and as a key crisis response measure, classes were mostly moved online. While navigating national public health settings, educators simultaneously had to innovate quickly: to keep our courses in operation, students learning, and quality pedagogy present. The author has been navigating a faculty-wide ‘dual-mode’ teaching policy at their home institution, to teach the Māori language. Dual-mode refers to teaching both in-person and online. Although dual-mode teaching has brought a significant amount of learning flexibility and innovation to the classroom, it also has disadvantages. This article attempts to document some of the positive and negative characteristics noted over two years of dual-mode teaching, from the position of intermediate Māori language classes. It concludes that although this kind of teaching is flexible and equitable, it is not of high quality, and contributes to poor proficiency outcomes for students. Teaching in this way could, however, be of a much higher quality, with support, resourcing and time. As we look to keep these more accessible teaching methods in the future, those additional aspects will be critical in designing classes that are not only equitable, dynamic, and in line with contemporary technological learning styles, but also of a standard that maintains quality language proficiency-building within our learners.

## Introduction

The COVID-19 Global Pandemic changed the landscape of higher learning, seemingly overnight, and possibly forever. Much of the world has endured a months-long battle with the virus, and educators worldwide quickly moved into online teaching through the need to limit human contact, but keep access to learning available and present. We have managed to do so, albeit perhaps to a lower standard of quality than we are used to.

Aotearoa/New Zealand has had a different virus trajectory than most nations. A tough stance on restricting human movement in an initial 2020 lockdown bought enough time to eliminate the virus completely. A more relaxed stance in 2022 has required us to live with COVID-19. Therefore, rather than experience the constant ebb and flow of infection other countries have, Aotearoa has spent considerable time either without COVID restriction at all, or with the bare minimum of health measures in effect. We have had two 4-week lockdowns, and a period of 4 months when vaccine mandates were in effect. For the other twenty-one months, however, most of Aotearoa has been reasonably free of restriction in comparison to other nations.

The New Zealand Government has implemented two national public health measures during the pandemic. The first, a 4-level Government-ordered alert level system, allowed different regions of the nation to operate with an appropriate amount of restriction, depending on regional risk and exposure to the virus. Of the twenty-one months mentioned above, the Auckland Region was significantly more affected by lockdowns than other parts of the country, due to the exposure of the virus there. The second is a ‘traffic light’ setting, which has undergone several iterations to date, but importantly removes the highest restriction—a lockdown. At the time of writing, Aotearoa is in the orange-light setting—there are no Government-enforced vaccine mandates in place and very little restriction on movement or social distancing, beyond a mask in tight spaces.

While the national public health measures made a good deal of common sense for a nation, citizens were mostly left alone to create systems to manage public health measures. Senior Leadership teams within institutions had to plan for core business operations amid a national pandemic response, and for the first time, that meant taking pedagogical control of the classroom.

Due to our public health settings spanning between full national lockdowns, zero restriction, and everywhere in between, institutions in New Zealand did not need to plan for online-only learning. In the main, universities have tried to incorporate in-person learning elements alongside online teaching methods, so that classes (and people) could pivot in accordance with national health settings, regional health settings, and personal health needs.

At the author’s institution, a blanket requirement of ‘dual-mode’ learning and teaching was instituted in mid-2020. Under the dual-mode requirement, courses must be taught in-person, when it is safe to do so, as defined by national public health settings. However, courses must also be online—for learners who may be essential workers (those who can work in a lockdown), in a regional lockdown, overseas, or otherwise unable to be on campus and in class. This online element may be executed in a number of ways: synchronous teaching (classes are taught in-person and live online, simultaneously), hybridisation (some of the classes are taught in-person only, and other classses are taught online only) or asynchronous teaching (classes are pre-recorded and uploaded to a learning management system later). The final aspect of the dual-mode requirement is that any live element of the course must be recorded, for students who are unable to be present at the time of the class. Essentially, this means every class must be able to be taught asynchronously, regardless of the other teaching modes employed, such as synchronous or hybrid.

Dual-mode teaching has been an important part of overall pandemic course resilience, to enable our programmes of study to continue, no matter what. It has opened up access in some forms, and is has brought innovation to the classroom. It seems to offer something to an international market and, in general, it looks and feels resilient. For those reasons, it would be easy to claim that dual-mode is a good way to be teaching, and should remain post-pandemic. For language teachers though, dual-mode is an intensely tough space to navigate. It is imperative to have as much time as possible to teach in, and through, the language we are aiming to pass on. Real-time communication is key, and that means as much time as possible must be spent in real-time contexts. This is especially true above the beginner level classes, when language output is a key focus of a course. The avenues for doing that through dual-mode teaching formats are equitable, but lower in quality. The most recent scholarship concerning online language learning seems to suggest online language learners need entirely separate planning and teaching methods from in-person students, and so, using one to inform the other is not likely to be successful (Sari, 2020; Todd, 2020). Dual-mode, as it is for language teachers, seems to force these two environments together. A key concern for this paper then, is that dual-mode teaching ‘looks good’, but it has a myriad of pedagogical challenges for the classroom that are not yet documented in the literature. More institutions may look to this model, or their own versions of it, post-pandemic. It is critical, and timely, to provide some cautionary writing around dual-mode teaching, and to do so urgently. This paper attempts to provide that.

To begin, this article provides an account of the latest language and pandemic literature (restricted to 2020 at the oldest). Because most institutions of higher learning around the world have been operating primarily online, readers will see the most current research lends itself to online language teaching only. From here, the article moves into the current dual-mode requirements for teaching at the author’s own institution, and the tasks required simultaneously in order to execute all the pedagogical requirements for a class teaching the Māori language at the intermediate level. The innovation employed, and the advantages and disadvantages are noted in a ‘field-note’ manner. Some perspectives on dual-mode and its possible relationship to tertiary student engagement are offered.

The article concludes that although both in-person and online language teaching pedagogies can be successful, equitable and of high quality, the weaknesses of each learning method means that in dual-mode form, only equity is achieved. Each avenue requires full, dedicated planning, resourcing and teaching. Although it makes sense as an urgent pandemic response, retaining dual-mode teaching for languages would likely be a pedagogical mistake in the long-term. However, with dedicated planning, resourcing, and teaching, this teaching format has shown new innovation, and that is not to be ignored as we head into the future.

The implications of this article are most critical to university-level decision makers, and language teachers. For those setting blanket administrative requirements at the faculty or university level, post-pandemic considerations for dual-mode learning, or forms of hybridising, do need to be flexible enough for language teachers to navigate. Other non-language courses may not need such flexibility. Additionally, release time and dedicated language resourcing are desperately needed if dual-mode platforms are going to remain as a key part of university offerings in the future. For language teachers, a full range of access measures, pedagogical planning and support measures are needed to enact dual-mode if it is to reach the same quality of teaching previously available to students, and this has a large effect on workload, and how administrative aspects of the course (such as marking feedback) are handled.

## Technology Throughout the COVID-19 Pandemic

Face-to-face (F2F) language classrooms are the mainstay for language learning, but they can still benefit from technology use (Gao & Zhang, 2020). Online language courses, when executed well, are also just as successful as traditional F2F classrooms (Gacs et al., 2020; Moser et al., 2021). Online learning though, has weaknesses. Having already surveyed the literature available, Todd (2020) notes that “Baralt et al. (2016) suggest that online learning may be less effective than classroom learning as students are less engaged, and Tang (2019) argues that face-to-face learning is more effective than online learning for teaching pragmatics.” Ng (2020) also summarises a wide range of studies on the topic, and presents common issues of “technical issues such as sound quality”, a “lack of emotive/cognitive qualities”, that “conversation is divorced from the context of shared social and physical surroundings” (Trinder, 2015, p. 94, in Ng, 2020), and that there is a lack of “facial expressions and body language, which students consider a vital aid towards understanding”. These paralinguistic features, coupled with the full experience of being in the same room are aspects that online learning does not, in the main, seem to provide.

Online language learning also places the agency on students involved to participate, engage, and self-direct their learning. Gao and Zhang (2020) cover the real experiences of educators who went through the online transition during the pandemic. One of their participants notes that:

Online teaching is diametrically different from traditional classroom teaching [...] online teaching is conducted in a virtual space, in which face-to-face interaction can hardly be achieved with the same effect as in a physical classroom. Limited by the online teaching conditions, it is difficult to guarantee the full participation of students; the learning outcomes are closely related to self-management and metacognitive ability on the students’ part (Gao & Zhang, 2020).

In-person and online pedagogies are quite different, and that online teaching inserts a participation barrier between the student and teacher. It also becomes incumbent upon the learner themselves to organise themselves and be more independent outside of class time, and this is not always desirable. This is further indicated by another participant:

None of the online teaching platforms can guarantee instantaneous interaction between teachers and students, which subsequently brings negative effect on the teaching efficiency. Due to the time lost in the lengthening of teacher–student interaction, online teaching capacity is less than that of traditional classroom teaching and a lot of work needs to be assigned for the students to do after class. Second, as the teacher cannot see every student in online teaching, it is more difficult to monitor their learning, not to mention their after-class assignments (Gao & Zhang, 2020)

Here, the participant notes that interactions have too much of a lag for speech interactions, which is a barrier to teaching real-life communicative competency. Secondly, the quality of the time the class has together is reduced, therefore leaving more work to the students to do independently. Of course, if a student has opted for online lessons, they may have prepared for these eventuations. In the throes of a pandemic, they have not had time to consider those necessities, and may not be in a position to access them.

In addition to overall efficacy, even the platforms we use have been critiqued. Of Zoom, Kohnke and Moorhouse (2020) state:

it has limitations when contrasted to F2F lessons. For example, group discussions tend to take longer and are more challenging to monitor; students tend to be less willing to self-nominate themselves to respond to questions or provide opinions due to the lack of paralinguistic cues; with larger classes, it can be hard to observe learners’ engagement; and students can get ‘screen fatigue’.

Furthermore, there is also the issue of anxiety online brings to the language classroom. (Russell, 2020) calls on a plethora of research which points out that there is already a fear of “real or anticipated interaction” “negative evaluation” and “test anxiety” in language classes. In the online space, learners may enter an online language class for “anonymity”, expecting not to have to communicate with others.

So, whilst not aiming to discredit the use of technology (as it has its merits, and other studies have already pointed out its relative efficacy to F2F teaching), there are significant barriers online learning imposes upon learners and teachers. Students must be more independent, more ready to engage in work after class, are not having the same quality interaction as F2F situations, and gaining their participation is harder. It also raises significant anxiety barriers, in an already anxiety-inducing space. While these factors, overall, impact on the learning abilities of online students amidst a pandemic, they are the only way to get around the issue of distance so greatly needed by virus responses.

## ‘Dual-Mode’—In the Classroom Mid-Pandemic, Equitably

The most recent literature above examines online language learning in comparison to F2F learning, or focuses solely on online teaching only. This would lead me suggest that most educators (and scholars contributing to pandemic related literature) are largely considering F2F teaching only or online-teaching only in their pandemic responses. There is very little literature discussion around what happens when the two teaching are encountered together, simultaneously, in a dual-mode setting. Having the two modes operating at the same time brings one key difference to the post-pandemic context in Aotearoa, about which international settings may not be familiar.

Only two recent publications were found that speak to dual-mode, and both suggest that wrapping an online mode around an in-person mode can be a self-defeating way to teach language. Todd (2020) points out that “simply repackaging the same content and activities from the classroom to online is not enough and higher levels of technology use need to be applied.” Others have noted that if in-person language classes are merely replicated for online language learners, it is likely to be “unsuccessful” (Sari, 2020). Therefore, simply reduplicating in-person classes and pedagogies for online learners is unlikely to succeed. Conversely, that is exactly what dual-mode seems to demand—everything must be delivered to the same extent, in an equitable manner, at the same time.

The term ‘dual-mode’ thus pertains to each class containing one synchronous teaching mode (in-person or livestreamed), but is also asynchronous. In a dual-mode setting, students may attend class in-person, or take part in online learning. They make this choice for any given class, on any given day. If participating online, students may attend a livestreamed class on Zoom, or not attend at all, with the intention of catching up later via the recorded videos of class. It demands both contexts are executed simultaneously, with every pedagogical need and individual learning need prepared, for every student, for every class.

Dual-mode has been necessary. There are equity issues to take account of, and dual-mode caters to this well. Not every student is able to learn online. Since we have not been exclusively bound to isolation and complete social distancing (although those measures have been a part of our response, at times), that in-person component is important and expected. Conversely, not every student is able to return to campus. Students with low immunity, or who live with immuno-compromised people, should not feel they have to return and be around other people. Tertiary students in the country form a large population of the essential work force, were hit hard financially by COVID-19 with almost no Governmental support in comparison to others (Akuhata-Huntington, 2020; New Zealand Union of Students' Associations, 2020). Students may have had to take on more employment, to earn more for their household facing loss of income from other people. Some students are international, and unable to enter the country. Lastly, certain regions have entered into a full lockdown, and students have been there at the time. They are unable to leave for that time and return to campus, and thus self-transition to online learning when needed. Dual-mode accounts for these issues in an equitable way, as much as possible, by offering a way to join in on classes despite those difficuties. With that said, online learning itself has its own equity issues. It might be assumed that the more access there is to a class, the more equitable it is. This is not true: “Simply offering emergency remote instruction is no guarantee that students have the resources and capacity to learn” (Moser et al., 2021). The inequity of access to technology is an international issue (Gao & Zhang, 2020). Aotearoa has not escaped this either (Brown, 2021; Matear & AQA, 2021), and the inequity for Māori students is greater than others (Akuhata-Huntington, 2020; Metson & Roy, 2021). A dual-mode environment thus provides some inroads towards access and equity, but relies on other equity issues also being solved.

In terms of delivering a course, the online aspect of a dual-mode course can be delivered done a number of ways, through live-streaming, or through recording lectures for off-campus students to watch. For a non-language course, it is possible to see how a resilient, hight quality course could be created, reasonably quickly. Taught content filmed in the past could be updated, edited, and utilised to provide a similar quality, online version of what was happening in an in-person class. Coupling this with some live interaction would provide a sound, resilient dual-mode course that was at least of a similar quality to in-person teaching.

Language tuition, however, has its own unique pedagogical needs, and some of these options are less possible for equity or quality. Firstly, recorded lectures cannot teach two-way communication. While certain parts of a course can be recorded (such as grammar explanations), the critical parts of communicative competency need to be communicative, and that means live teaching. Secondly, no cohort of learners is ever the same—a high quality language classroom must respond to that dedicated cohort with each rotation of the course. Their individual language experiences and proficiencies, which are many and varied, cannot be accounted for until those students are encountered by the teacher. Language trauma too, is important for Māori language contexts (Te Huia, 2020) and needs to be considered for each individual student personally, and in real-life. There are tears and struggles in the classroom, and learning the language to a competent communication level requires settling those trauma issues in students affected by that. Therefore, communicative competency is not theoretical, cannot be assessed through any means except real-life interaction, and cannot be taught without mostly real-life interaction, with the currently enrolled students. Live interaction is a necessity of the pedagogy.

## An Educator’s Experience with Dual-Mode

Having now defined dual-mode and commented on equity, it is now an appropriate time to document the author’s delivery of language courses to intermediate speakers of Māori over an eighteen month period during 2020–2021, in a dual-mode setting. Eight courses in total have been delivered, with 48 h of instruction for each course. Each course is one hour and fifty minutes long. Below is an outline of how dual mode classes are operated by the author, with as much detail as possible. This consists of approximately four semesters worth of personal evaluation.

As a offer in a field-note style, the key limitation is that the following is just one author’s perspective, and this part of the paper lacks any kind of empirical study. However, with the concern that more universities around the world will begin to seek dual-mode teaching methods of this kind, and solidify them in faculties without thinking carefully about language instruction, it feels more valuable to disseminate such notes with some urgency, rather than complete a long-term study on dual-mode learning, which would absolutely confirm or refute the perspective presented here.

### Room Setting

In the language classroom is a row of tiered seating, which seats about 100 students. One or two large projector screens face the students, and project the computer and overhead projectors situated on the lecturn. This lecturn is placed at the front of the room, and it houses a touch-screen panel that controls imaging, sound and room lighting. There is one teacher in the room who is responsible for instruction, and all technological needs for the class.

In terms of visual capture, there is one static camera in each room. In some cases, the camera is a professional device pointing towards the lecturn and projector screens. It is placed high up on the ceiling, in the back of the room. It is not possible to capture (and thus teach) body language at this height and distance. Lecture slides also need not be captured by this camera—they are provided on the learning management system, and the institution’s video capture software also captures the computer’s own screen displaying the slides. It seems unnecessary to capture them via camera as well, but this kind of capture may be relevant to other disciplines and their courses.

In other classes, there is no professional camera, but a residential web camera placed on the lecturn’s computer screen. The teacher generally places this to face the in-person students to foster inclusivity with those online, and so the online students can see other students interacting or asking questions. A slideshow presentation accompanies each lecture, but it seems more pertinent for students at home to see what the class is doing, rather than watch either the lecture slides or the lecturer’s face for the full two hours. Having said that, because there is nothing to capture the instructor’s body, face and movement, the online students do not get to observe the face, hand and body gestures of a proficient speaker, and so, are not able to learn those skills. To try and cater to this, on occasion, the lecturer will move to the students seating and lecture from there.

There are multiple options for audio capture—the lecturn has a microphone, and there are three roaming options—lapel microphone, hand-held microphone, and throwable soft cube with a microphone inside. Feedback issues can arise when these roaming options are used and in-person students are also using Zoom with their devices unmuted (this is explained below, in the pedagogies section). Otherwise, this works well.

### Equitable Curriculum Planning

As mentioned above, students can opt to learn synchronously (in-person or via livestream) or asynchronously (lecture recordings), as they like, and as public health measures allow. From an equity perspective, students should be able to see everything that happens in a lecture, and do every activity that is set, regardless of where they are learning from. For those reasons, all of the various ways in which students might arrive to a class needs to be accounted for. It is normal to have anywhere between one and twenty students online, and between five and thirty students in-person. There have been occasions where no one is online, but no instances as yet of having no students in-class. Regardless, the lesson cannot rely on any certain number of students attending through either format. All group activities need to be planned for all eventuations of class make-up, so they can respond immediately to the class on the day, as it arises. This needs to be planned well in advance, and often, posted on the learning management system (LMS) in advance, for online users. As language classes must be dynamic and responsive, much of the class content is student-created, and additions to the learning material are made frequently throughout class. At the end of class, the earlier posted material on the LMS needs to be updated again, to reflect what the students achieved that day. All of these things help to create inclusion and equity for the class as a whole, but create considerable stress for the sole instructor, who still carries all the other aspects of teaching a course (marking, administration, meetings, and so forth).

### Kinaesthetic Learning Tools

Although dual-mode planning offers the chance for innovation, it also restricts what can be done in the in-person language classroom. This raises quality questions. For example, no kinaesthetic learning activity that would benefit in-person learners only can be used. If online learners cannot see it, touch it, or replicate it themselves without first buying the product required—that is inequitable. Therefore, all board games, language cards and the like have been disregarded in this dual-mode environment. The teacher has created some inclusive language games, such as code-cracking activities to open an electronic, encrypted file placed on the LMS. These received great participation at first, but the exciting novelty they brought wore off quickly. For any object being used as a language teaching tool, the teacher must try to pre-empt what all students may have on hand—a pen, or themselves, for example. Beyond that, it is difficult to teach with kinaesthetic tools, and they are rarely used.

Other physical learning tools such as the whiteboard are also not used—they are not within decent view of a camera and do not reproduce well on a digital screen. Therefore, they are not inclusive enough of online learners. The Zoom whiteboard function is used as a digital replacement, and the overhead projector is used for a hand-written replacement. Both are visible to in-person learners. So long as it is setup correctly, the video capture software captures it also, and Zoom users or those watching a lecture recording will be able to see any notes made.

### Communicating, and Being Communicated With

Audio is inevitably a critical feature of dual-mode language teaching. The teacher cannot stray too far from a microphone, and neither can students. Students learning in-person are sometimes asked to use throwable microphone cubes, in order to be heard better by students at home. At times, students enjoy the ability to throw around an inanimate object. At other times, it is an imposition to be coerced into speaking into a microphone—it is as if your language learning (and any mistakes made) are a performance for all to hear. At times, in-person students are asked to use their own devices to log in as a Zoom learner, to enable their microphones. This increases the exposure of the in-class learners to the online learners. This works well, although multiple active microphones in the same place can cause feedback, amplify general class noise, and cause private conversations to be caught by the lecture recording—as long as students are on Zoom, they are being recorded by the LMS recording software.

All lessons are live-streamed in a two-way format, over Zoom— it must be two-way, so students at home can communicate with others in class. Other seminar platforms are one-way, and so, would not be appropriate. For activities, in-person students are sometimes asked to also be active on the live-stream, so as to be more inclusive.

Communication between the teacher and learner is restricted in a dual-mode setting. The teacher must also respond to chat messages, and be able to audibly hear, and speak, to everyone participating at home. The teacher tries not to move around too much, so as not to fall outside of the shot of a camera, or microphone. This has placed more of a teacher-student barrier on in-person learners, as there is less chance to move around the classroom and respond to individual students and small groups. When moving around the room, the lecturer is mostly absent from view on the screen, although they can be heard through the use of an additional roaming microphone. Disappearing from view can leave online students feeling excluded or bored, and they can remove themselves for that lesson. In online learning, there can be a demand for immediacy and instant satisfaction—the teacher needs to be available for that. If the teacher cannot do that because they are busy with an in-person group, the desire to wait is often too long for online learners, who expect that instant gratification. Since all students know the entire class is captured, there seems to be a temptation to log-off when this happens, with the intention of perhaps checking the lecture recording later (this is also commented on below, in the student engagement section).

Online learners are able to write on the Zoom chat, and can be audibly heard by the class as a whole. While this raises their ability to participate inclusively, it also raises the anxiety of online learners who do not always want to ask questions online, where things might be seen by the class as a whole. Different things to mitigate this have been trialled, such as carrying headphones for setting up an ad hoc, private Zoom session midclass with a student, or minimising the Zoom camera screens before class. This also has merits and failings—headphones work well, but provide another barrier of visual distance for in-person learners—the fact the instructor’s ears are bound to listening elsewhere is a communicative barrier for a spoken language. Minimising the zoom screen causes online students to become ‘ghosted’ from the class—the teacher cannot see when they want to visually raise a question, and in-person students cannot interact with them visually.

### Asynchronous Learning

For this teaching mode, all classes are recorded and uploaded to the LMS. The teacher does not use Zoom’s function to record for these students. With Zoom, there are two options for recording—it must either be local to that computer, or to the cloud. Local Zoom recordings aren’t possible, as they would end up on the lecture theatres resident computer—this would be a student privacy breach as the computers are used by any number of people in a day. Cloud recordings of the two-hour class length can take several hours to upload to Zoom, and then must be moved manually to the LMS. Thankfully, the LMS at the institution carries its own video capture integration. Classes can be scheduled to begin and end automatically, and are uploaded instantly to the LMS. This works very well for administration. Disadvantages of this are that there is very little editing available on the software, so the captures really are just a representation of what has happened in the session, and that is not always visually appealing to the student. The ideal time for a lecture recording should not exceed twenty minutes (Özkara, 2021), but there is not enough time for a teacher to edit and optimise longer classes into smaller segments solely for correspondence learners. Students are left with an unedited capture of the class spanning approximately 120 min, and that is not very stimulating for learners. This is perhaps not equitable, but at least the entire course’s content is available.

Asynchronous learners must be able to see everything that happens in a lecture, and do every activity set, in their own time, and must be able to understand each message the teacher sends clearly. For the benefit of correspondence learners, the teacher must consistently monitor every word they say for any items of speech that would be ambiguous to a correspondence viewer. For example, they cannot say ‘see this example here, yes that one’. The message must be: ‘can everyone see this example I’m circling with my mouse right now’, or other such example. The teacher must foretell in the moment what it might be like to watch the recording back, and to learn everything that is said. As a result, the language taught in dual-mode classes is often less emotive and more robotic. This provides some loss of quality to other learners, who could otherwise learn those things.

Some students have taken up the offer of asynchronous study as an opportunity to study entirely by correspondence. At the author’s institution, all courses are designated as dual-mode on course outlines, and in the course selection information when enrolling. Correspondence is thus set at this point as a possible option. For some students, this has enabled them to stay connected to their learning, while perhaps having to work or take care of family members throughout the pandemic. At the author’s institution too, attendance requirements are not widely accepted because of the inequity that brings. Therefore, not many courses can *require* students to attend. The author supports this, entirely. Having said that, without some kind of ‘hook’ to suggest attendance is important, then it is possible students will be less likely to engage and participate, or learn the material. For most courses, a certain amount of ‘cramming’ is probably possible – for a language, however, the most important factor a student can bring is the hours spent in class. As a result of these things, it is a key concern that the notion of dual-mode itself dissuades students from engaging with the course, and that has a negative affect on their achievement.

Correspondence is an entirely different learning style for languages, where asynchronicity is concerned. It is merely one-way communication—the lecturer recording talking at them. It becomes incumbent on the learner to find their own avenues for two-way communication practise, if they are not participating in synchronous avenues offered. If working and supporting family throughout a pandemic is the reason students are entering correspondence learning, there will be no time to find those avenues, and spend time reclaiming those lost hours.

How instutitions think about asynchricity, and how students think about the same, matters. Potts and Stebletsova (2021) point out that “[o]ne of the potential issues with online learning is that the expectations of the students and the tutor can differ, so taking the time at the beginning of the course to align these expectations is beneficial to both the learner and tutor”. If that choice has already been made at enrolment, and the student indeed has already been encouraged to study by correspondence, there is almost a permission given to disengage—they may not even enter the classroom to hear the teacher’s own expectations.

This is particularly worrying for students encountering university for the first time. First-time students are transitioning into university life through a dual mode lens for the first time. Cameron et al. (2021), in their study, noted younger students share higher levels of anxiety, isolation, and confusion about the parameters of university study. Having spent 18 months in this teaching environment, this author is positing that this is true for students in Aotearoa. Because asynchronosity is an option, there are students who have not yet attended a class that other students are attending highly, in another format. Petitioning the importance of attendance cannot be allayed without first meeting those students in a class, and are much harder to unravel if they have already decided not to come, and catch up later through a recording. For language courses, there is no catching up on lost language time, without more language time.

In summary, it is the feeling of this author that dual-mode teaching for languages brings a much higher amount of work to enact, but a different quality of teaching into the classroom. Operating together, none of the formats seem to be of a quality they could be if taught independently. While it brings resilience to course continuation, and equity of access to many different kinds of students, there are also trade-offs for all learners, and things they do not get in their learning experience. Because the teacher must plan for any number of students to utilise any of the learning methods, for any lesson, planning for simpler and more superficial classes is a consistent result. More basic tasks are prepared to ensure classes have that consistency, and there is less option to provide interesting and inspiring lessons that help to grow a passion within students for language.

The difficulty of asynchronous learning provisions, and correspondence, are also a key concern. For languages there is no replacement for hours spent engaging in the language, and it is so important that students are encouraged to understand this fact as they enrol. It is also important instructors have the ability to signal these pedagogical necessities, and make these decisions, for the achievement and learning of their students.

## Lessons for the Future

There is no reason to shy away from online teaching in any respect, nor dual-mode teaching. However, on the other end of the pandemic, and as language teachers clear themselves from the chaos and look to the future integration of technology to the tertiary classroom, there are some critical things to plan for. Dual-mode, hybridisation, and all manner of synchronous and asynchronous teaching formats will likely stay on in the calendars of institutions, and they should. Gacs et al. (2020) note: “Attention to instruction in F2F, online, and hybrid formats will undoubtedly continue to be a necessity, with special attention to adaptability, flexibility, and quality instruction remaining at the center.” The question is what these things – flexibility, adaptability and quality instruction—look like in language courses. Importantly, the author adds equity to that list.

One fundamental way to improve on this is giving language teachers the flexibility themselves, to choose how they deliver online components of the class. As Todd (2020) points out:

It is important to understand what happened in the shift to online learning and why this happened to be able to identify directions for the future which could increase the likelihood of successful online learning and to identify those initiatives which have already provided success. This issue is especially important for a subject like English language which relies on teacher student interaction to a greater extent than most other subjects at universities.

The same is true for all language classrooms. The aspect underpinning all of this is quality, and the time it takes to produce quality for language classes in dual-mode environments. MacIntyre et al. (2020) state that “there has been an expectation that teachers will simply carry on and do their best by adapting, adjusting and continuing to aim for effective communicative language teaching using a range of online resources. How realistic it is for educators to meet these demands has not been at the forefront of most (any) policy decisions, and the stress being produced is something of an afterthought—if it is considered at all.” A real option here would be to release pedagogical control to teachers and programmes, even if the institution instructs that online elements need to be present, so that teachers may move within the realm of online blending however they need to, in order to avoid that stress.

As well as relinquishing the classroom’s pedagogical needs, there are support mechanisms greatly needed for language teachers. These have already been established in the literature. If dual-mode teaching is to remain eternally, the reality of teaching a language class requires more dedicated thinking than pandemic, or normal university life, allows currently. González-Lloret (2020) notes that “It is important to have realistic expectations about the working load that online courses produce for teachers”, and that “… a well‐developed and methodologically sound curriculum, based on language acquisition research findings, has to be the foundation of any language course. Todd (2020) further adds that “in the case of the pandemic the shift to online learning was so sudden that trialling different versions of the innovation was impossible.” Outside of the pandemic, is where we must be able to do this innovation, but that will take time. Courses continue, and students continue to arrive and depart. There is little time to spend on thinking, planning, and sourcing efficient and high-quality ways to deliver for students, and the consistent turn of normal university life does not allow for that. Gacs et al. (2020) posit that such educators need “release time or other compensation”, because there is a huge amount of work involved in establishing quality online courses. At their institution, some staff “were given a course release per semester during the development years and are now given one‐course release per academic year for maintenance and training.” This length of time is supported by others, such as Moser et al. (2021), who state that a quality online course requires “six to nine months of planning”. If we are to establish quality online, or dual-taught language courses that are resilient, equitable and quality-based, we will need that time. If not time, perhaps additional staff with permanent, long-term roles could be available, and those positions available before a course has to run. Those staff could be part of that planning, before they are present in the classroom to help cater to multi-mode delivery. Perhaps too, courses could be hybridised, with a staff member allocated to each format. “We cannot reset 2020, but it can be argued that we can reset ourselves with updated cognitions and upgraded knowledge and skills of information technology literacy” (Gao & Zhang, 2020). This author is happy to reset, to innovate, and to plan around the dual-mode problems outlined here, but that will require assistance and time, as we look past the pandemic to resilient, equitable and quality classes that employ technology for achievement’s sake, and communicative competency.

## Data Availability

Not applicable.

## Code Availability

Not applicable Introduction:
